# Orally Available Selective Melanocortin-4 Receptor Antagonists Stimulate Food Intake and Reduce Cancer-Induced Cachexia in Mice

**DOI:** 10.1371/journal.pone.0004774

**Published:** 2009-03-19

**Authors:** Philipp Weyermann, Robert Dallmann, Josef Magyar, Corinne Anklin, Martina Hufschmid, Judith Dubach-Powell, Isabelle Courdier-Fruh, Marco Henneböhle, Sonja Nordhoff, Cesare Mondadori

**Affiliations:** Santhera Pharmaceuticals (Switzerland) Ltd., Liestal, Switzerland; New York State Institute for Basic Research, United States of America

## Abstract

**Background:**

Cachexia is among the most debilitating and life-threatening aspects of cancer. It represents a metabolic syndrome affecting essential functional circuits involved in the regulation of homeostasis, and includes anorexia, fat and muscle tissue wasting. The anorexigenic peptide α-MSH is believed to be crucially involved in the normal and pathologic regulation of food intake. It was speculated that blockade of its central physiological target, the melanocortin (MC)-4 receptor, might provide a promising anti-cachexia treatment strategy. This idea is supported by the fact that in animal studies, agouti-related protein (AgRP), the endogenous inverse agonist at the MC-4 receptor, was found to affect two hallmark features of cachexia, i.e. to increase food intake and to reduce energy expenditure.

**Methodology/Principal Findings:**

SNT207707 and SNT209858 are two recently discovered, non peptidic, chemically unrelated, orally active MC-4 receptor antagonists penetrating the blood brain barrier. Both compounds were found to distinctly increase food intake in healthy mice. Moreover, in mice subcutaneously implanted with C26 adenocarcinoma cells, repeated oral administration (starting the day after tumor implantation) of each of the two compounds almost completely prevented tumor induced weight loss, and diminished loss of lean body mass and fat mass.

**Conclusions/Significance:**

In contrast to the previously reported peptidic and small molecule MC-4 antagonists, the compounds described here work by the oral administration route. Orally active compounds might offer a considerable advantage for the treatment of cachexia patients.

## Introduction

Cachexia is among the most debilitating and life-threatening aspects of cancer. It is associated with anorexia, fat and muscle tissue wasting, and a progressively decreasing quality of life [1]. The presence of cachexia is a predictor of poor survival. Up to 80% of patients with cancer develop cachexia before death, and in over 20% of all cases cachexia is responsible for the death of the patient [2], [3]. At the moment of diagnosis, about 80% of patients with gastrointestinal cancers and 60% of patients with lung cancer have substantial weight loss. In general, patients with solid tumors (with the exception of breast cancer) have a higher frequency of cachexia [4]. Cachexia is a predictor of poor outcome not only for cancer patients but also in various other chronic diseases [5]–[8].

Even though the exact nature of the underlying mechanisms remains largely unknown, it is evident that cachexia represents a metabolic syndrome caused by a complex interaction between the tumor and the host. Cachexia is characterized by major metabolic abnormalities and maladaptations: Food and therefore energy intake is reduced, resting energy expenditure is often increased and catabolism is accelerated [3]. The emerging view is that cachexia represents the clinical consequence of a chronic, systemic inflammatory response and many of the physiological, metabolic, and behavioral changes of cachexia have been found to be tightly regulated by cytokines. For example, cytokines have been found to be involved in depletion of skeletal muscle [9], signaling the synthesis of acute-phase proteins [e.g. 10], regulation of energy expenditure [e.g. 11], and decreased food intake [e.g. 12].

One mechanism by which the cytokines (and other appetite regulating molecules such as leptin) can induce anorexia is via the regulation of pro-opiomelanocortin (POMC) expression [13]. POMC is a precursor molecule for important endogenous peptides such as adrenocorticotropin (ACTH), α-, and β-melanocyte stimulating hormone (α-MSH and β-MSH), γ-Lipotropin and β-Endorphin which are produced via cleavage by tissue specific enzymes. POMC neurons are mainly located in the arcuate nucleus of the hypothalamus. POMC neurons are considered to have major regulatory functions in food intake and energy expenditure. It is assumed that these effects are predominantly mediated by α-MSH, a 14 amino acid peptide with appetite inhibiting effects [14].

Alpha-MSH, the endogenous ligand at the MC-4 receptor, and other agonists at the MC-4 receptor have been found to inhibit food intake, increase energy expenditure and reduce body weight. Inversely, disruption of melanocortin signaling with agouti related peptide (AgRP) or small molecule MC-4 receptor antagonist treatment or deletion of the receptor led to increased food intake and reduced energy expenditure [15]–[18]. Accordingly, in the context of creating a treatment option for cachexia patients it was speculated that interruption of this signaling pathway could eventually reduce the progression of cachexia [19], [20].

SNT207707 and SNT207858 are the results of a major effort to find selective, potent and orally active MC-4 receptor antagonists. SNT207707 binds to the MC-4 receptor with an affinity of 8 nM and shows a more than 200-fold selectivity vs. MC-3 and MC-5. SNT207858 is a 22 nM MC-4 antagonist with a 170-fold selectivity vs. MC-3 and a 40-fold selectivity versus MC-5 [21], [22]. In order to assess the potential usefulness of these compounds for the treatment of cachexia we evaluated their acute effects on feeding during the light phase in healthy mice. Moreover, we investigated the effects of repeated treatments on possibly clinically relevant parameters in a mouse model of cancer cachexia.

## Materials and Methods

### General

SNT207707 and SNT207858 were synthesized in the Medicinal Chemistry Department at Santhera Pharmaceuticals (Switzerland) Ltd. All animals were held under standard laboratory conditions (21±1°C, 40–60% humidity) with 12 hrs of light per day (05:00 to 17:00 h) and free access to food (2018S Teklad Global, Harlan, CH) and tap water. All compounds were freshly dissolved and administered by oral gavage at the indicated doses in a volume of 10 ml/kg of 10% Hydroxypropyl-β-cyclodextrin (Acros Organics, Geel, BE) in 100 mM saline solution unless otherwise noted. Syngenic C26 colon adenocarcinoma cells (CLS Cell Line Service, Eppenheim, DE) were cultured in serum free medium (Quantum 263; PAA Laboratories, Linz, A) before implantation in animals. All animal experiments were approved by the governmental authorities (permission numbers BL246, BL356).

### Light Phase Food Intake

Six weeks old female NMRI mice (RCC Ltd.; Füllinsdorf, CH) were randomly assigned to the various experimental groups and the vehicle controls. They were housed in groups of 3 mice per cage. After at least one week of acclimatization in the experimental room, each animal's weight was determined and compound or vehicle was administered at appropriate concentrations 5 hrs after lights on. Then, food consumption of each cage group was recorded by weighing of the food hopper over a period of 4 hrs. Food intake was then determined as the difference in food hopper weight at the beginning and end of the 4 hrs following administration of the compound or the vehicle. Since the amount of food taken over 4 hrs by single animals was close to the limit of detection, we decided to rely on mean food amounts taken per cage (i.e., per 3 mice), whereby each treatment group consisted of n = 6 cages. The measured values from each cage were normalized to 100 g body weight (BW). Previous experiments indicated an excellent correlation between food intake and reduction of food weight.

### C26 adenocarcinoma-induced cachexia model

Six week old male BALB/c mice (Harlan, The Netherlands) were used. The animals were single housed, and after a one week adaption period randomly assigned to the experimental or control groups. For the tumor implantation the mice were anaesthetized with 200 µl of ketamine/xylazine. Approximately 1×10^6^ C26 cells suspended in phosphate buffered saline were then implanted unilaterally in the flank of the mice by subcutaneous injection. The non-tumor controls underwent the same procedure but received PBS injection only (n = 9 per group). Starting on the first day after tumor implantation, compounds or vehicle were administered orally in 5 ml/kg 1–2 hrs before the onset of the dark phase. Food intake and body weight were recorded daily. Lean and fat mass of each animal were determined by MRI relaxometry (EchoMRI-500, Echo Medical Systems, Houston, TX, USA) prior to tumor inoculation (day 0) and at the end of the experiment after removal of the tumor (typically day 15).

### Brain and plasma concentrations *in vivo*

Twelve weeks old male CD-1 (Hilltop Lab Animals, PA, USA) mice were dosed by gavage with either SNT207707 or SNT207858 at 60 mg/kg (n = 9 per compound). At 1, 3, and 6 hrs post-dose, 3 mice from each compound group were euthanized with CO_2_. Blood was collected by cardiac puncture, plasma was isolated immediately and then kept on dry ice until analysis. Brains were removed, rinsed with saline solution and stored at −80°C until analysis. Levels of the compound were determined by HPLC followed by mass spectrometry (MS/MS). Areas under the curve (AUC) of plasma and brain levels were computed from measured levels at 1, 3 and 6 hrs post-dose. Brain/plasma ratios were then calculated based on the AUCs. To assess the integrity of the blood-brain-barrier all animals were co-dosed with 10 mg/kg Atenolol. Brain levels of Atenolol close or below limit of detection indicated that the blood-brain-barrier was intact.

### Statistics

Differences between groups were compared with (repeated) analysis of variance (ANOVA) with Dunnett's post hoc test where applicable. Kaplan-Meier curves for occurrence of cachexia were plotted with GraphPad Prism and statistically compared using the Mantel-Cox test. All test were carried out 2-tailed with α = 0.05. All results are given as means±SEM.

## Results

### Light Phase Food Intake

**Figure 1:**
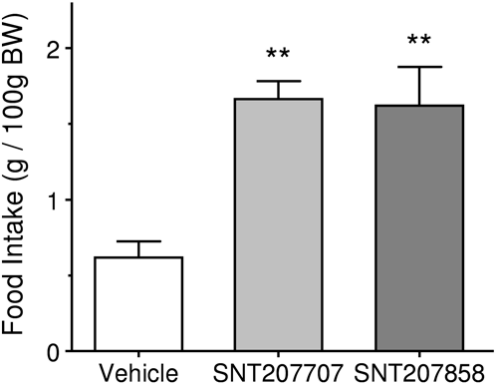
The effects of s.c. injection of Vehicle (open bar), 20 mg/kg SNT207707 (light grey bar) and 20 mg/kg SNT207858 (dark grey bar) on light phase food intake in healthy mice. Animals were held in groups of three. Food intake per cage (n = 6 cages/group) was recorded over a period of 4 hrs starting after injection of compound. Each bar represents mean±SEM. Statistical difference vs. Vehicle ** p<0.01.

A single *subcutaneous* injection of 20 mg/kg of either SNT207707 or SNT207858 distinctly increased food intake of the mice (ANOVA p<0.001). The increase was statistically highly significant for both compounds (p<0.01). The amount of food taken during the four hours observation period was roughly 3-fold the amount taken by the vehicle treated controls. Figure 1 depicts the results graphically.

**Figure 2:**
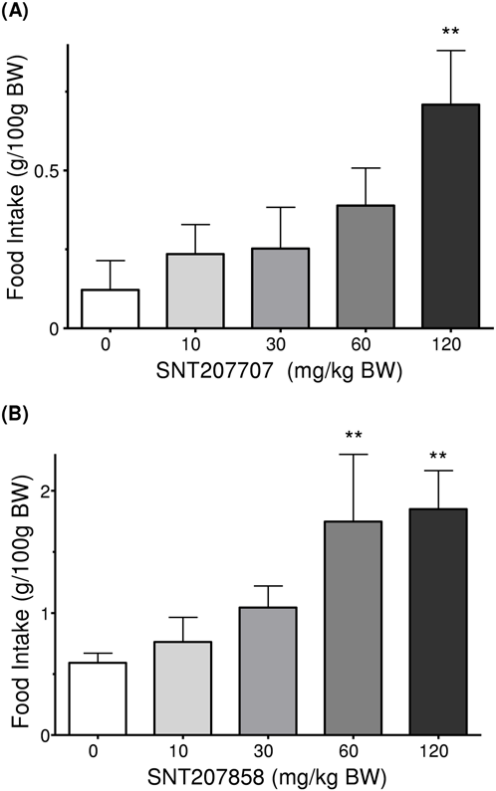
The effects of p.o. administration of (A) SNT207707 and (B) SNT207858 on light phase food intake in healthy mice. Animals were held in groups of three. Food intake per cage (n = 6 cages/dose) was recorded over a period of 4 hrs starting after administration of compound. Each bar represents mean±SEM. Statistical difference vs. Vehicle ** p<0.01.

In line with the results via the subcutaneous administration route, a single *oral* treatment by gavage with either SNT207707 or SNT207858 dose-dependently increased the food intake (ANOVA: SNT207707 p<0.01 and SNT207858 p<0.05). Single dose comparisons revealed statistically significant increases in food intake at 60 and 120 mg/kg for SNT207858 (p<0.01), and 120 mg/kg for SNT207707 (p<0.01). The amount of food taken during the four hour post-treatment observation period was roughly up to four times the amount taken by the vehicle control group. Figure 2 is a graphical representation of the data produced.

### C26 adenocarcinoma-induced cachexia model

**Figure 3:**
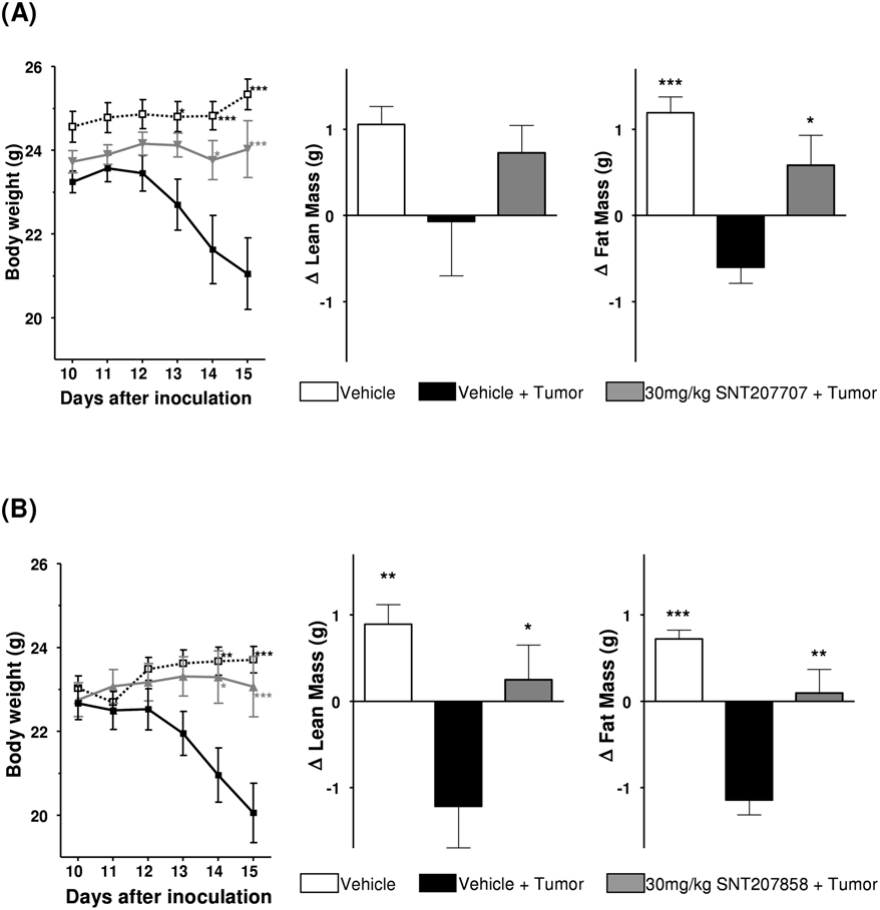
The effects of (A) SNT207707 and (B) SNT207858 in C26 tumor bearing mice. Left panels: Mean body weight development of Vehicle control (open squares), Vehicle+Tumor control (closed squares), and 30 mg/kg (A) SNT207707 and (B) SNT207858 (grey triangles) group (n = 9 each). Middle and right panels: Difference in lean body mass and fat mass between day of tumor inoculation (day 0) and end of experiment (day 15). Each value represents mean±SEM. Statistical difference vs. Vehicle+Tumor * p<0.05, *** p<0.001.

Once daily oral administration of both compounds SNT207858 and SNT207707 starting the day after tumor implantation significantly reduced the tumor induced weight loss. The outcomes of the two experiments were quite comparable: In both experiments, the tumor was palpable around day 4 after inoculation. Similarly, in both experiments the vehicle-treated tumor controls stopped gaining weight around day 11 and began to show weight loss on day 13. In both experimental series, the compounds almost completely prevented weight loss, and the average body weight of the tumor bearing compound treated groups was significantly higher than that of the Vehicle+Tumor group. Figure 3 (left panel) depicts the results graphically.

**Figure 4:**
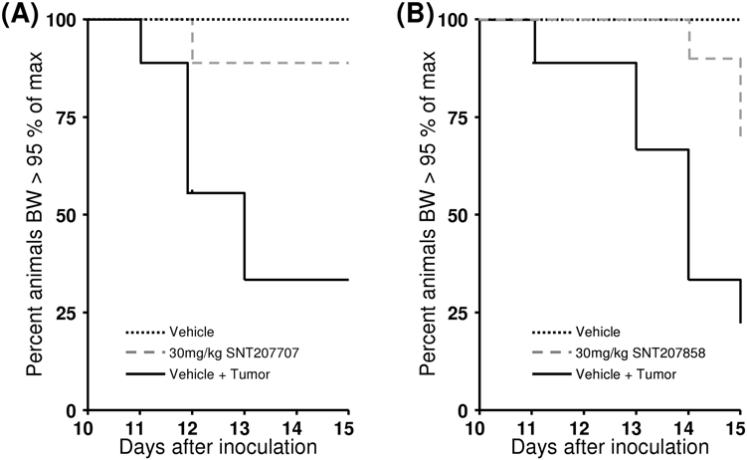
The effects of C26 tumor on development of cachexia (defined as loss of more than 5% of body weight (BW) after inoculation of tumor cells) in mice. (A) Left panel: Kaplan-Meyer plot for Vehicle control (dashed black line), Vehicle+Tumor control (black line), and SNT207707 30 mg/kg (grey line) group (n = 9 each). (B) Right panel: Kaplan-Meyer plot for Vehicle control (dashed black line), Vehicle+Tumor control (black line), and SNT207858 30 mg/kg (grey line) group (n = 9 each). Note: Statistical comparison between Vehicle+Tumor and treatment groups was significant.

Although both compounds did greatly ameliorate symptoms of cachexia, tumor growth in both compound groups was not altered compared to Vehicle+Tumor group. Tumor weights in mice treated with SNT207707 (1.03±0.07 g) and SNT207858 (1.11±0.09 g) were not different from those in the corresponding control groups (1.17±0.06 g and 1.19±0.05 g, respectively). It has to be noted that even though all inoculated animals developed a tumor, not all tumor bearing mice became cachectic, i.e. lost more than 5% of body weight in the course of the experiment. According to this definition, cachexia was not observed in 2 of 9 and 3 of 9 animals of the Vehicle+Tumor control groups whereas 8 out of 9 in the SNT207707 and 6 out of 9 mice in the SNT207858 treated group, respectively, did not show cachexia. Kaplan-Meier analysis of the onset of cachexia revealed a statistical difference between the SNT207707+Tumor (p<0.05) and SNT207858+Tumor (p<0.01) groups each compared to respective Vehicle+Tumor controls (Figure 4).

Both compounds were found to have distinct effects on body composition. The non tumor vehicle controls showed increases in fat mass and lean body mass whereas the vehicle treated tumor animals showed distinct losses of both, fat mass and lean body mass. Both compounds partially counteracted the cancer induced changes, i.e. no loss but even a slight gain of fat and lean body mass was observed. Figure 3 (middle and right panels) depicts the results graphically.

### Brain and plasma concentration in vivo

After oral application, CD-1 mice showed significant levels of both compounds in plasma and brain (Table 1). The calculated maximal concentrations in brain are 580 nM for SNT207707 and 110 nM for SNT207858.

**Table 1:** Peak plasma and brain levels and 0-6-hr plasma and brain AUC of MC4-R antagonists SNT207707 and SNT207858 in CD-1 mice after oral application (n = 3 per group).

Compound	SNT207707	SNT207858
Dose [mg/kg]	60	60
Plasma peak [nM]	1960	1520
Plasma AUC [nM·h]	6373	4405
Brain peak [nM]	580	110
Brain AUC [nM·h]	2307	437
Brain/plasma ratio	0.36	0.10

Note: Brain/plasma ratio is calculated from area under the curve (AUC).

## Discussion

Our results show a significant enhancing effect of SNT207707 and SNT207858 on food intake over a 4 hrs observation period after oral administration to mice. These robust orexigenic effects confirm published findings showing that endogenous peptidic or small molecule MC-4 receptor antagonists enhance food intake in healthy animals [23]–[25]. The significance of the compound's enhancing effects on food intake is further supported by the fact that in our experiments the drug-induced feeding happened during the inactive light phase, where, under normal (non-stressed) conditions, food intake is low [26]. Moreover, the animals had 24 hrs free access to food and water, i.e. they were not fasted. Accordingly, the drug effects might be interpreted in terms of an acute appetite-enhancing effect.

The results of the tumor induced cachexia experiments are equally clear. SNT207858 and SNT207707 showed distinct anti-cachectic effects at the dose used, i.e. both compounds almost completely blocked the tumor induced loss of body weight, and also had positive effects on body composition. Both, loss of lean body mass and loss of fat mass was reduced. These anti-cachectic effects were not due to any anti-tumor effects since determination of the tumor growth and weight did not indicate any effect of the compounds on the tumor. Even though all of the tumor implanted mice did develop a tumor, and all tumors developed from a single batch of tumor cells, there were single animals in the Vehicle+Tumor group showing no cachexia. The reason for the lack of cachexia is not known, but it has to be assumed that there might have been animals not showing cachexia in the drug treated groups as well. Nevertheless, the number of non-cachectic mice in the Vehicle+Tumor group is significantly smaller than in the treatment groups for both compounds (Figure 4), further supporting a beneficial effect of the treatment. In the absence of any useful marker to differentiate between spontaneous and drug related absence of cachexia and in order not to bias the outcome of the experiment all animals were included in the statistical evaluation when comparing means for body weight or body composition.

The observed effects of SNT207858 and SNT207707 in the C26 cancer cachexia model are in line with previous findings by us and other groups reporting effects of MC4-R antagonism in animals with cancer induced anorexia. For example, MC-4 receptor blockade through the central administration of AgRP or the MC-3/4 antagonist SHU-9119 increased food intake in models of cancer-induced anorexia [20], [27]. Earlier results indicated that subcutaneous administration of the small molecule MC-4R antagonist ML00253764 was efficacious in reducing cachexia in animals implanted with either a colorectal tumor [28] or Lewis lung carcinoma [23]. Consistent with these findings, intra-peritoneal administration of NBI-12i, another MC-4R antagonist, ameliorated cachexia in mice with implanted Lewis lung carcinoma and uremia. This small molecule antagonist did not only reduce tumor induced anorexia but also increased lean body mass [24]. Finally, there is a recent summarizing report on a whole series of compounds from Neurocrine Biosciences described to have positive effects after intra-peritoneal administration in cancer cachexia models [29].

In the light of the *a priori* hypothesis an important question concerns the involvement of central neural circuits in the observed appetite enhancing effects, or in other words: are the brain levels achieved with the compounds high enough to be causally involved in the effects? SNT207707 has an IC50 of 8 nM (binding) and 5 nM (function) on the MC-4 receptor [21]. The approximate brain concentration in the mouse was calculated to be 580 nM (Table 1). Accordingly, from the concentration point of view nothing speaks against a central effect. The same holds true for SNT207858, i.e. the maximal brain concentration is about 110 nM [22], and thus, considering the IC50s of 22 nM (binding) and 11 nM (function), above the concentration required for central effects (Table 1).

Facing the clear-cut effects on food intake the next question is how far these effects *per se* can account for the observed anti cachexia effects. Considering any potential interpretation of our effects in terms of an appetite enhancing effect immediately leads to a comparison of the active dose range in the light phase food intake and in the cancer cachexia model. Accordingly, a significant beneficial effect in the cancer induced cachexia experiment was detected at 30 mg/kg p.o. (Figure 3). This effect includes reduction of body weight loss and the positive effects on lean body mass and fat mass. At the very same dose, in the light phase food intake experiment, only a slight trend (if anything) towards increased food intake could be detected (Figure 2). In support of an appetite based interpretation many aspects contributing to a possible dose mismatch can be brought up. First, the light phase food intake experiments have been done with our standard laboratory strain (NMRI) whereas, due to the need for a syngenic tumor and mouse line, BALB/c mice have been used for the cancer cachexia experiments. Moreover, the regulation of food consumption is based on an extremely complicated, highly cross-linked network involving the brain, autonomous nervous system, gastro-intestinal tract, and adipose tissues involving a variety of neurotransmitters, hormones and peptides [30]–[32]. Cachexia is the result of a profound functional disturbance of these networks; presumably by pro-inflammatory cytokines (see above). Accordingly, the drug effects are superimposed onto a fundamentally different background activity and therefore even more profound changes, not limited to shifts in sensitivity towards compounds, could have been expected.

An important aspect, speaking against an interpretation solely in terms of an appetite enhancement is the fact that measurements of food intake in our cancer cachexia experiments provided non conclusive results, i.e. food consumption was extremely variable between days of the experiment and no clear effect towards increased food intake could be identified (data not shown). Moreover, there is no doubt that physiological changes in cachexia are not just involving a lack of appetite. The cachectic patient is distinctly different with regards to metabolic adaptations. In healthy subjects, caloric deprivation induces physiological adaptations to conserve energy and to protect tissue. In contrast, cancer patients show maladaptive responses resulting in inappropriate high energy expenditure despite low caloric intake [3]. Accordingly, therapeutic approaches aiming at both, increasing food intake and reducing energy expenditure are expected to be therapeutically superior to approaches aiming solely at food intake. This particular aspect might make the MC-4 receptor antagonist approach therapeutically most promising since the effects of an interaction with the melanocortin signaling pathway are expected to have consequences broader than just a modulation of appetite. As previously mentioned, α-MSH, the endogenous agonistic ligand at this receptor was found to have a dual action, i.e. to reduce food intake [14] and also to increase energy expenditure [33]. In turn, agouti related protein, the endogenous inverse agonist to this receptor was observed to block the MC-4 receptor and, inversely to the action of α-MSH, increase food intake and reduce energy expenditure [34]. Accordingly, via the MC-4 receptor, two key aspects of cachexia can possibly be treated and the anti-cachectic effects of our compounds in the C26 cancer model might be based on such combination effects. Thus, in future experiments, effects on energy expenditure have to be shown experimentally, e.g., by indirect calorimetry.

Even though our results are completely in line with the literature there is one major difference: In contrast to the above mentioned experiments with peptidic and small molecule MC-4 antagonists, the compounds described here work by the oral administration route. In the light of patient care, orally active compounds might offer a considerable advantage. Moreover, it has to be noted that SNT207707 and SNT207858 represent two different, chemically unrelated compound classes, MC-4 receptor blockade being the only common denominator.
